# Contact coverage, content of care and inequality in antenatal care and routine immunisation in rural Uttar Pradesh, India: a repeated cross-sectional analysis of household surveys and direct observations of community health sessions

**DOI:** 10.1136/bmjph-2026-005227

**Published:** 2026-11-02

**Authors:** Esther Shoemaker, Emma Sandstrom, Ravi Prakash, John Anthony, Fernando C Wehrmeister, Aruni Tennakoon, Lorine Pelly, Bidyadhar Dehury, Amit Kumar Agrawal, Vasanthakumar Namasivayam, Dhanunjaya Rao Chintada, Shajy Isac, Ramesh Banadakoppa Manjappa, James Blanchard, Marissa L Becker

**Affiliations:** 1 University of Manitoba, Winnipeg, Manitoba, Canada; 2 India Health Action Trust, Lucknow, Uttar Pradesh, India; 3 Universidade Federal de Pelotas, Pelotas, Brazil

**Keywords:** Community Health, Health Services Accessibility, Sociodemographic Factors, Pregnancy, Maternal health

## Abstract

**Introduction:**

Community-based primary care aims to reduce geographic and opportunity–cost barriers in rural settings, yet population trends in coverage, content and education-related inequality are poorly described. In rural Uttar Pradesh (UP), monthly community health sessions (Village Health and Nutrition Days (VHNDs)) provide integrated antenatal care (ANC) and routine immunisation (RI). We describe long-term trends in coverage, content and education-related inequality, with direct observation of session readiness.

**Methods:**

We analysed repeated cross-sectional rural UP household survey data from National Family Health Survey (NFHS)-3 (births 2001–2006), NFHS-4 (2011–2016) and NFHS-5 (2018–2020). Outcomes included any and community-level ANC/RI, content-qualified ANC (Antenatal Care Quality (ANCq) ≥8), full RI among children aged 12–23 months and ultrasound use and caesarean birth as facility-dependent comparators. Education-related inequality was quantified using the Slope Index of Inequality and Concentration Index (CIX). Direct observations of 61 133 VHND sessions (2022–2023) described input readiness and observed ANC component delivery.

**Results:**

Between 2001 and 2019, any ANC increased from 51.3% to 94.8% and content-qualified ANC from 3.3% to 53.3%. Any RI remained high throughout; full RI increased from 20.8% (2004) to 71.2% (2019). Gains were largest among women with no formal education. Relative inequality narrowed for every indicator and fell close to zero for services deliverable at community level (CIX 0.502 to 0.059 for ANCq, 0.269 to 0.018 for full RI), while remaining substantial for caesarean birth (0.394 to 0.257). Sessions showed high blood pressure and haemoglobin testing capacity and strong availability for key antigens, with lower but improving urine testing and measles-rubella vaccine supply.

**Conclusions:**

Content-qualified ANC and full immunisation improved markedly, with the largest gains among the least educated women and relative inequality narrowed for every indicator except caesarean birth. Where community-deliverable services have converged across education groups, the remaining gap lies in facility-dependent care. Further gains likely depend on referral, transport and surgical capacity rather than extending first-contact outreach.

**WHAT IS ALREADY KNOWN ON THIS TOPIC**

**WHAT THIS STUDY ADDS**

- Across two decades in rural Uttar Pradesh, content-qualified antenatal care rose from 3.3% to 53.3% and full routine immunisation from 20.8% to 71.2%, with the largest gains among women with no formal education, whose full immunisation coverage rose from 13.9% to 67.6%. Education-related inequality narrowed markedly for these content-qualified antenatal and immunisation services, mostly delivered through monthly community health sessions, but remained substantial for facility-dependent services (ultrasound use and caesarean birth), highlighting divergent education-related inequality trajectories across the primary care continuum. Direct observations of 61 133 community health sessions, to our knowledge the largest reported assessment of this kind, found that these sessions were generally equipped with the blood pressure, haemoglobin, urine testing and vaccine inputs required by programme norms.

**HOW THIS STUDY MIGHT AFFECT RESEARCH, PRACTICE OR POLICY**

- Education-related gaps have largely closed for services deliverable at a monthly village session, but not for caesarean birth, so further gains depend on referral, transport and surgical capacity rather than on extending first-contact outreach. Monitoring based on contact indicators alone can overstate progress and should track care content and completeness.

Community outreach extends contact with antenatal and immunisation services in rural settings, but contact-based coverage indicators do not capture whether recommended care was actually received.

## Introduction

 Delivering primary care to rural and hard-to-reach populations requires service delivery models that reduce geographic and informational barriers while reliably delivering evidence-based service content. The WHO’s guidance on community health worker programmes emphasises that community-based delivery can extend coverage of essential services, but that programme impact depends on system supports such as training, supervision, data systems and supply chain reliability.^1,2^

In India, the Village Health and Nutrition Day (VHND) was established as a monthly, fixed-site outreach session intended to connect communities to the public health system and deliver a convergent package of reproductive, maternal and child health and nutrition services.^3^ VHNDs are typically organised at Anganwadi Centres and delivered by a frontline team anchored by an auxiliary nurse midwife (ANM), with mobilisation and support from Accredited Social Health Activists (ASHAs) and Anganwadi Workers.^3^ Hereafter, we refer to VHNDs as community health sessions. The normed service package includes pregnancy registration and antenatal care (ANC), routine childhood immunisation (RI), vitamin A supplementation, growth monitoring/weight plotting and selected counselling and prevention messages. By bringing services closer to households on a predictable day, community health sessions are designed to reduce time and travel costs and support continuity of preventive care. Uttar Pradesh (UP), India’s most populous state, remains a priority setting for public health systems strengthening. Despite national progress, UP contributes a large share of India’s maternal deaths and state-level estimates indicate persistently elevated maternal mortality risk relative to many other regions.^4,5^ These patterns underscore the need for delivery strategies that improve the coverage and content of essential services for women and children across socioeconomic strata.

ANC and RI are among the high-priority services delivered through community health sessions because they prevent avoidable morbidity and mortality and support care continuity across the maternal–child continuum. However, contact-based coverage metrics, such as four or more ANC visits, can overstate progress when the clinical content of care is incomplete.^6^ The ‘quality-coverage gap’ is well documented, and contact indicators do not necessarily reflect receipt of evidence-based interventions. Measurement approaches increasingly combine contact with content, including the validated content-qualified ANC indicator (Antenatal Care Quality (ANCq)), which summarises receipt of key recommended components rather than counting visits alone.^7^ ANCq has been applied to India at national level within multicountry analyses and used to describe socioeconomic inequality in ANC across low- and middle-income countries.^8^ Related content-based quality measures have since been applied across all 36 Indian states and union territories.^9^ In this study, quality is defined operationally as the availability of essential inputs and the receipt of evidence-based service components, rather than provider skill or clinical outcomes. For immunisation, quality similarly extends beyond whether a child is recorded as vaccinated. Reliable service delivery requires consistent session functioning, vaccine availability, safe administration and adequate cold chain and logistics.^10^ In rural India, research links public-sector facility quality and readiness to immunisation outcomes and to socioeconomic gaps in coverage, suggesting that equity gaps may reflect both demand-side barriers and system quality deficits.^10^ National and state-level analyses show that immunisation inequality has declined over time, yet education-related and wealth-related gaps persist, with maternal education repeatedly associated with differential uptake.^11,12^

Evidence on the functioning of community health sessions in UP is limited and predominantly district-specific but suggests variable fidelity to the normed package. In a peer-reviewed assessment in Hardoi district, for example, ANC and vaccination were offered in most observed sessions, yet session cancellation, vaccine shortages and incomplete delivery of other services were common.^13^ Meanwhile, long-run analyses in UP indicate that education-related inequality in antenatal contact coverage has declined over recent decades, while persisting at finer programme-delivery layers such as blocks and individual community health worker catchments.^14^ Those analyses tracked whether women had contact with antenatal services; they did not examine whether the content of that care improved, did not extend to RI and did not compare services deliverable in the community with services that require a higher-level facility. Both of those findings are consistent with the inverse equity hypothesis, which predicts that strengthened interventions may first benefit advantaged groups, with equity improving as coverage expands.^15^

UP has strengthened implementation of community health sessions under the ‘Chhaya Integrated Village Health Sanitation and Nutrition Day’ approach, which aims to deliver a defined reproductive, maternal, newborn and child health and nutrition service package on a fixed day each month.^16^ Yet long-term trends in the coverage, care content and education-related inequality of ANC and RI over this period of strengthening and scale-up have not been described. Therefore, this study examines long-term trends in the coverage and care content of ANC and RI among rural women and children in UP, services typically delivered at monthly community health platforms, examines how these trends are distributed by maternal education and household wealth and contrasts them with antenatal ultrasound use and caesarean birth as facility-dependent comparators not delivered at community health sessions. We describe care content using complementary measures of service availability/readiness and service uptake/content, and quantify differences by maternal education and household wealth using absolute and relative summary measures, consistent with WHO-recommended principles for health inequality monitoring.^17^

## Materials and methods

This study used a repeated cross-sectional design to examine trends in ANC and RI coverage, care content and socioeconomic inequality among rural women and children in UP, India. Analyses draw on multiple rounds of nationally representative household survey data capturing births occurring between 2001 and 2020, complemented by direct observation data on service availability and readiness at community health sessions collected between 2022 and 2023.

### Patient and public involvement

Patients and the public were not involved in the design, conduct, reporting or dissemination of this research.

### Setting and data sources

Trends in ANC and RI service uptake were assessed using data from the women’s questionnaire of the three most recent rounds of the Indian National Family Health Surveys (NFHS)-3,^18^ NFHS-4^19^ and NFHS-5.^20^ For trend analyses, maternal indicators were assigned to the calendar year of birth and are reported for 2001–2005, 2011–2015 and 2018–2019. RI indicators were estimated among children aged 12–23 months at interview and assigned to the child’s calendar year of birth (2004–2005, 2013–2015 and 2018–2019). Pooled cross-round analyses, including the inequality estimates, use the full birth cohort for each round, while the annual trend series exclude right-censored terminal years. The NFHS employs a stratified, multistage cluster sampling design and is representative at the national and state levels. The present analysis was restricted to rural areas of UP. We used routine programme monitoring data derived from direct observations of community health (VHND) sessions conducted between January 2022 and November 2023 across rural UP. Observations were restricted to those collected using the same version of the observation checklist. Each observation recorded session functioning and the availability of essential ANC and RI inputs at the start of the session, including the presence and functionality of blood pressure (BP) measurement equipment, haemoglobin (Hb) testing capacity, urine testing supplies and vaccine stocks and diluents. The observation system used a non-repetitive sampling approach within 6-month cycles, intended to ensure that each subcentre/ANM catchment delivering services in rural areas was observed once per cycle. Survey data were used to assess long-term trends and inequalities (2001–2020), while observation data provided postsurvey evidence on session-level input availability and observed delivery of selected ANC components (2022–2023).

### COVID-19 considerations

Routine service delivery in UP was disrupted during the COVID-19 pandemic in 2020.^21^ To avoid conflating pandemic-related disruptions with longer-term trends, births (for ANC analyses) and childbirth-years (for RI analyses) occurring after April 2020 were excluded from the pooled cross-round analysis and the annual trend series are reported to 2019, the last birth year with substantially complete cohort capture. Because each survey applies a fixed lookback window to a moving interview date, births in the final year of fieldwork can only be reported by women interviewed late in the field period and the number of births contributing to each annual estimate falls sharply in those years.

### Study population/participants

The study included rural women aged 15–49 who had given birth within 5 years preceding NFHS-3 and NFHS-4, and within 2 years preceding NFHS-5, as well as their children aged 12–23 months at the time of each survey. As a result, the ANC analytic sample represents rural women’s most recent singleton live births within the relevant lookback period for each survey round, and maternal indicators were analysed by the birth year. For RI indicators, analyses were restricted to rural children aged 12–23 months at the time of interview and RI indicators were analysed by the child’s calendar year of birth.

### Measures

Socioeconomic inequalities were assessed primarily using maternal education, categorised as no education, 1–5 years, 6–9 years and 10 or more years of schooling. Education was selected as the primary stratifier because it is a stable measure of socioeconomic position, consistently captured across survey rounds, and serves as a marker for health literacy, household resources, social position and capacity to navigate the health system.^22^ Parallel analyses were conducted using household wealth indices.

#### Self-reported service uptake

Content-qualified ANC was assessed using the ANCq score, a comprehensive measure that combines basic care coverage, skilled provider care and selected clinical content indicators,^7^ with a score of 8 or higher indicating content-qualified ANC. The ANCq score ranges from 0 to 10 and assigns points for contact with a skilled provider, initiation of first-trimester care, number of ANC visits and receipt of key service components, including BP measurement, Hb testing, neonatal tetanus protection and urine testing.^7^ Higher scores indicate more complete receipt of the recommended ANC components. Studies have validated the ANCq’s robustness across diverse countries, exemplified by the consistent inverse relationship between the ANCq and neonatal mortality.^7,8^ To describe uptake of care delivered at community health sessions, we additionally measured community-level ANC as the proportion of women who reported receiving any ANC at a subcentre, an Anganwadi/Integrated Child Development Services (ICDS) centre or a village clinic by an ANM. For ANC indicators, responses coded as ‘don’t know’ or missing were retained in denominators but not counted as having received the component (ie, not counted in numerators), consistent with Demographic and Health Surveys (DHS) indicator guidance.^23^ Ultrasound use during pregnancy and caesarean section birth were examined separately as facility-dependent utilisation indicators not delivered at community level. These are measures of service use; because the surveys do not capture clinical indication, they cannot be interpreted as measures of appropriate, needed or high-quality care.

For child health, the primary RI outcome was receipt of full RI, defined consistently across NFHS rounds as receipt of BCG, three doses of oral polio vaccine (OPV), three doses of a diphtheria-tetanus-pertussis equivalent vaccine (DPT) and a measles-containing vaccine (recorded in NFHS as measles/measles-mumps-rubella (MMR)/measles-rubella (MR) depending on round). In NFHS-5, where pentavalent vaccination (Penta) was explicitly recorded, children were considered complete if they received either three doses of Penta or three doses of DPT. Newer antigens introduced in later survey rounds (eg, rotavirus, fractional-dose inactivated poliovirus vaccine(IPV)) were not included in the primary trend definition to ensure comparability across rounds. We measured community-level RI as the proportion of children who received any vaccination at a subcentre/ANM or an Anganwadi/ICDS centre. Vaccination status was based on home-based records when available and, otherwise, on maternal report. When neither source indicated receipt for a given dose (including ‘don’t know’/missing), the dose was classified as not received, consistent with DHS guidance.^23^ Of the survey outcomes, only community-level ANC and community-level RI indicate where care was received; ANCq and full RI are population coverage indicators that span all providers.

#### Observed service availability and service uptake

Using the direct observation dataset, we assessed whether each observed community health session met predefined minimum input criteria at the start of the session. Minimum quantities were drawn from programme norms for VHND functioning and reflected the practical requirement to provide services to attending women and children, including open-vial policy considerations for vaccines. ANC readiness was assessed for (1) a BP measurement device recorded as present and functional; (2) Hb testing capacity recorded as present and functional and (3) urine testing readiness, defined as at least 10 urine test strips in stock together with an appropriate arrangement for urine sample collection. For RI readiness, we assessed the availability of key antigens aligned with survey outcomes (BCG, OPV, DPT-equivalent/Penta and measles-containing vaccine recorded as MR in programme data). Availability thresholds were defined as at least 10 doses per antigen, operationalised using recorded quantities of closed and open vials, with open-vial quantities converted to dose equivalents using the programme’s conversion rule and rounded up as required. Sessions were also required to have diluent available for vaccines that require reconstitution (BCG and MR). Because MR is supplied in five-dose vials, at least two vaccine vials together with at least one diluent vial were required. Antigen-specific vial sizes are given in the note to figure 1. We also described observed delivery of selected ANC components among pregnant women attending observed sessions by computing, for each session, the proportion who received BP measurement, Hb testing and urine testing during the observation. Observed RI administration to children was not available in the monitoring dataset and was therefore not analysed.

**Figure 1:**
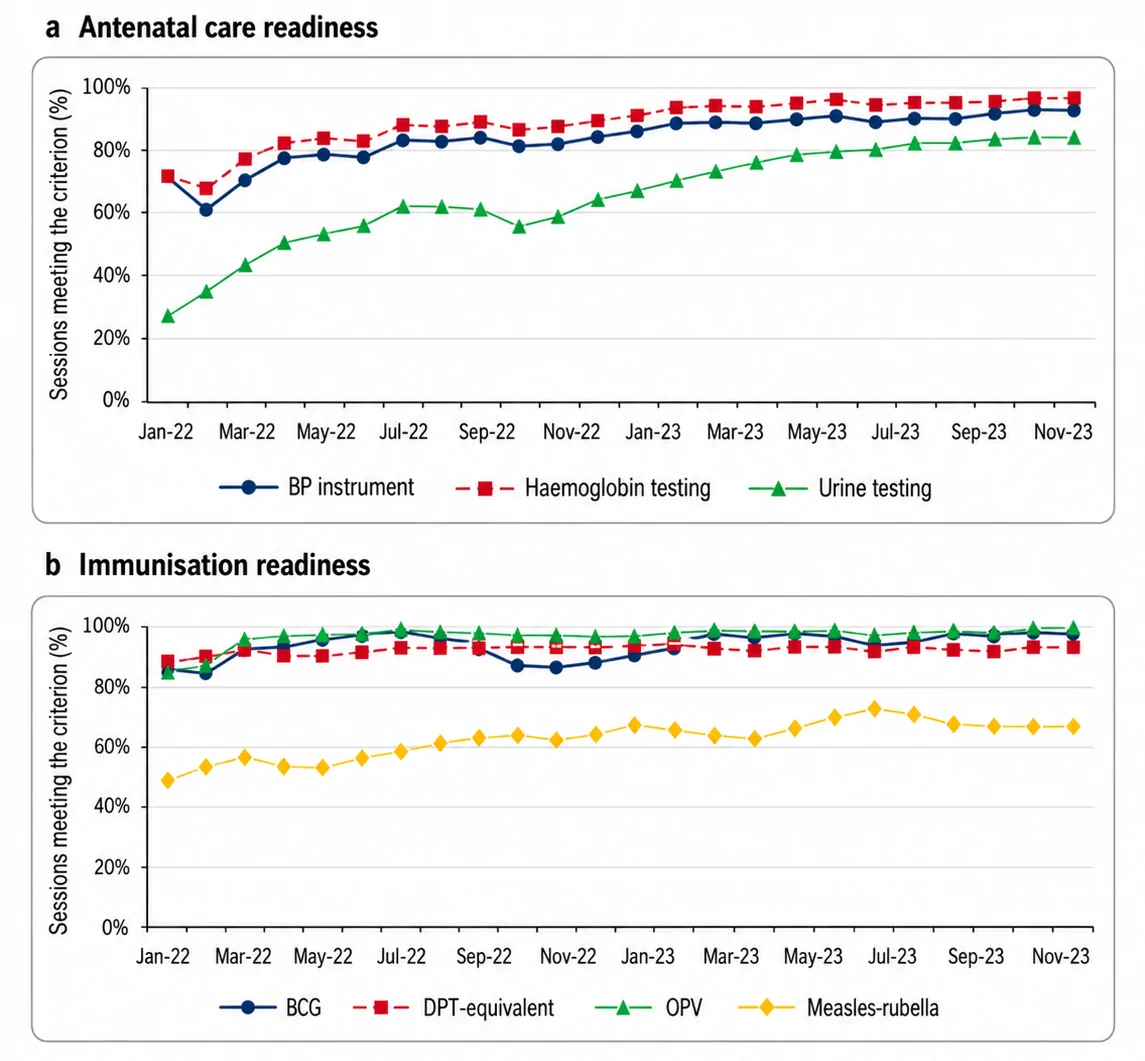
Readiness of community health sessions (VHNDs) to deliver antenatal care and routine immunisation services in rural Uttar Pradesh, India: direct observations, January 2022 to November 2023. Values are the percentage of observed sessions meeting each criterion, by month. (a) Antenatal care readiness: blood pressure (BP) instrument, a BP device (manual or digital) available and functioning; haemoglobin testing, a Sahli’s kit, Tallqvist scale or digital analyser available and functioning; urine testing, at least 10 urine test strips in stock and an appropriate place for urine collection. (b) Immunisation readiness: at least 10 doses of each antigen, counting closed vials at their full labelled size (20 doses for OPV, 10 for BCG, pentavalent and DPT, 5 for measles-rubella) and open vials as half. BCG and measles-rubella are freeze-dried and additionally required at least one diluent vial; because measles-rubella is supplied in 5-dose vials, two vaccine vials were needed to reach the 10-dose threshold, whereas one 10-dose BCG vial was sufficient. DPT-equivalent was met by either pentavalent or DPT. DPT, diphtheria-tetanus-pertussis; OPV, oral polio vaccine; VHNDs, Village Health and Nutrition Days.

### Statistical analysis

Trends in ANCq scores, full RI coverage and individual service components were examined across survey rounds and by maternal education level. Socioeconomic inequalities were quantified using the Slope Index of Inequality (SII) and Concentration Index (CIX). Within each survey round, women (for ANC indicators) and children (for RI indicators) were ordered by maternal education and assigned a fractional rank at the midpoint of the cumulative weighted population share of their education category; the same procedure was applied to household wealth quintiles for the parallel analyses. The SII assessed absolute differences across the educational gradient and the CIX assessed relative inequality. Positive CIX values indicate higher coverage among women with higher educational attainment. All NFHS analyses accounted for the complex survey design using sampling weights, cluster and strata variables to generate rural UP-representative estimates. Inequality indices (SII and CIX) were computed using education rank variables derived within each survey round and estimated with survey design corrections.

Analysis of observation data focused on describing the proportion of community health sessions meeting predefined criteria for service availability and readiness, as well as observed ANC service uptake among attending women. All analyses were conducted using SAS V.9.4 and Microsoft Excel V.16.

## Results

### Participants

After exclusions, analyses included 3045 births to rural women in 2001–2006, 22 154 in 2011–2016 and 8669 in 2018–2020, together with 839 children aged 12–23 months in the RI cohort for 2004–2005, 5956 in 2013–2015 and 2794 in 2018–2020 (table 1). Across survey rounds, most women were aged 20–35 and married. Educational attainment increased substantially, with the proportion reporting no education declining from approximately 70% in 2001–2006 to less than 30% in 2018–2020; the education strata therefore represent different shares of the population in each round. Parity declined over time, with most women reporting one or two children by 2018–2020. Programme monitoring captured direct observations of 61 133 community health sessions conducted between January 2022 and November 2023 (2022: 28 493; 2023: 32 640), and direct observations of service inputs and care processes among 542 780 pregnant women attending these sessions (2022: 234 533; 2023: 308 247).

**Table 1:** Characteristics of rural ANC and routine immunisation analytic cohorts in Uttar Pradesh, India, across National Family Health Survey rounds 3, 4 and 5

Characteristics	Rural women in ANC cohort			Women with children (12–23 months) in RI cohort		
	2001–2006 (n=3045)	2011–2016 (n=22 154)	2018–2020 (n=8669)	2004–2005 (n=839)	2013–2015 (n=5956)	2018–2020 (n=2794)
	n (%)	n (%)	n (%)	n (%)	n (%)	n (%)
Age, years						
<20	226 (7.4)	414 (1.9)	201 (2.3)	65 (7.7)	119 (2.0)	31 (1.1)
20–35	2468 (81.1)	19 569 (88.3)	8112 (93.6)	708 (84.4)	5465 (91.8)	2676 (95.8)
*>*35	351 (11.5)	2171 (9.8)	356 (4.1)	66 (7.9)	372 (6.2)	87 (3.1)
Education						
None	2123 (69.7)	9840 (44.4)	2529 (29.2)	597 (71.2)	2497 (41.9)	620 (22.2)
1–5 years	316 (10.4)	3113 (14.1)	1196 (13.8)	82 (9.8)	866 (14.5)	357 (12.8)
6–9 years	351 (11.5)	4164 (18.8)	1841 (21.2)	90 (10.7)	1190 (20.0)	630 (22.5)
10+ years	255 (8.4)	5037 (22.7)	3103 (35.8)	70 (8.3)	1403 (23.6)	1187 (42.5)
Wealth quintile						
Q1 (poorest)	1061 (34.8)	8252 (37.2)	2715 (31.3)	299 (35.6)	2151 (36.1)	818 (29.3)
Q2	908 (29.8)	6132 (27.7)	2609 (30.1)	257 (30.6)	1687 (28.3)	764 (27.3)
Q3	607 (19.9)	3994 (18.0)	1657 (19.1)	167 (19.9)	1111 (18.7)	541 (19.4)
Q4	356 (11.7)	2382 (10.8)	1094 (12.6)	85 (10.1)	643 (10.8)	406 (14.5)
Q5 (wealthiest)	113 (3.7)	1394 (6.3)	594 (6.9)	31 (3.7)	364 (6.1)	265 (9.5)
Parity						
One child	547 (18.0)	5458 (24.6)	2727 (31.5)	149 (17.8)	1577 (26.5)	870 (31.1)
Two children	577 (18.9)	5749 (26.0)	2609 (30.1)	182 (21.7)	1698 (28.5)	924 (33.1)
Three children	540 (17.7)	4387 (19.8)	1625 (18.7)	161 (19.2)	1147 (19.3)	503 (18.0)
Four or more children	1381 (45.4)	6560 (29.6)	1708 (19.7)	347 (41.4)	1534 (25.8)	497 (17.8)
Other characteristics						
Hindu	2495 (81.9)	18 617 (84.0)	7414 (85.5)	680 (81.0)	4967 (83.4)	2372 (84.9)
Scheduled caste/tribe	837 (27.5)	6290 (28.4)	2572 (29.7)	233 (27.8)	1705 (28.6)	885 (31.7)
Other backward caste	1602 (52.6)	12 267 (55.4)	4748 (54.8)	434 (51.7)	3293 (55.3)	1461 (52.3)
Currently married	3011 (98.9)	21 915 (98.9)	8601 (99.2)	833 (99.3)	5897 (99.0)	2765 (99.0)
Living with spouse	2391 (78.5)	17 753 (80.1)	6912 (79.7)	664 (79.1)	4777 (80.2)	2246 (80.4)
Miscarriage, stillbirth and/or abortion	716 (23.5)	5261 (23.7)	1332 (15.4)	180 (21.5)	1297 (21.8)	475 (17.0)
Death of a child	1013 (33.3)	4800 (21.7)	1301 (15.0)	246 (29.3)	971 (16.3)	309 (11.1)
Health insurance coverage	NA	339 (1.5)	456 (5.3)	NA	65 (1.1)	177 (6.3)
Chronic health condition(s)	51 (1.7)	524 (2.4)	380 (4.4)	11 (1.3)	112 (1.9)	99 (3.5)

ANC, antenatal care; NA, not applicable.

### Trends in ANC and RI coverage

Figure 2 shows sustained increases in ANC uptake at community health sessions across the survey periods, rising from 24.8% in 2001 to 54.1% in 2019. Receipt of any ANC became nearly universal (51.3% to 94.8%), and receipt of content-qualified ANC (ANCq ≥8) increased from 3.3% to 53.3%. For child health services, receipt of RI at community health sessions increased from 27.8% in 2004 to 75.1% in 2019. Receipt of any RI was already high in 2004 (97.3%) and remained consistently high (97.6% in 2019), while full RI increased from 20.8% to 71.2%. Figure 3 shows improvements across ANC components, including first-trimester care (18.6% to 60.3%), BP measurement (7.2% to 88.4%), Hb testing (3.9% to 85.8%) and urine testing (6.9% to 83.6%). Ultrasound use increased from 3.3% to 84.1%, and caesarean section births from 1.0% to 12.9%. Among women attending observed sessions, BP measurement rose from 77.6% in 2022 to 88.8% in 2023, Hb testing from 80.0% to 92.3% and urine testing from 51.3% to 76.0%. Among RI components, coverage of BCG (59.2% to 82.5%) and MR (35.8% to 66.7%) increased considerably between 2004 and 2013. By 2019, most rural children had received BCG (93.4%), a DPT-equivalent third dose (Penta3: 80.7%) and a measles-containing vaccine (MR: 84.5%). Third-dose OPV coverage was 76.3% in 2019, below its 2004 level of 87.6%.

**Figure 2:**
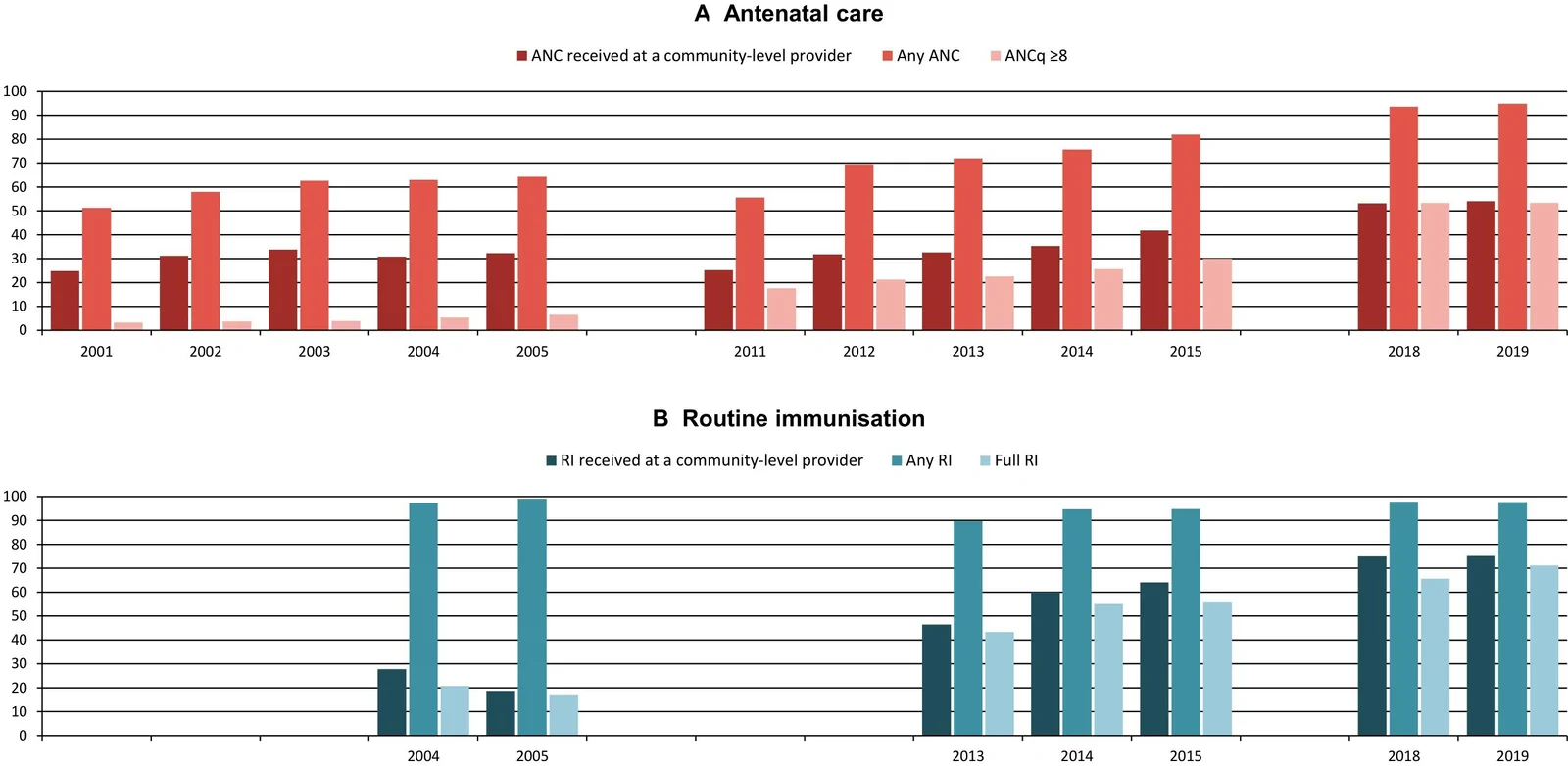
Coverage and content of antenatal care (ANC) and routine immunisation (RI) in rural Uttar Pradesh, India.(A) Antenatal care: ANC received at a community-level provider, any ANC, and content-qualified ANC (ANCq ≥8). (B) Routine immunisation: RI received at a community-level provider, any RI, and full RI. Estimates are from the National Family Health Survey rounds 3, 4 and 5, restricted to rural Uttar Pradesh. Antenatal indicators are among women with a live birth in the stated year (most recent live birth only); immunisation indicators are among children aged 12–23 months at interview, plotted by the child’s year of birth. ANCq ≥8 denotes a content-qualified antenatal care score of at least 8. ANCq, content-qualified antenatal care.

**Figure 3:**
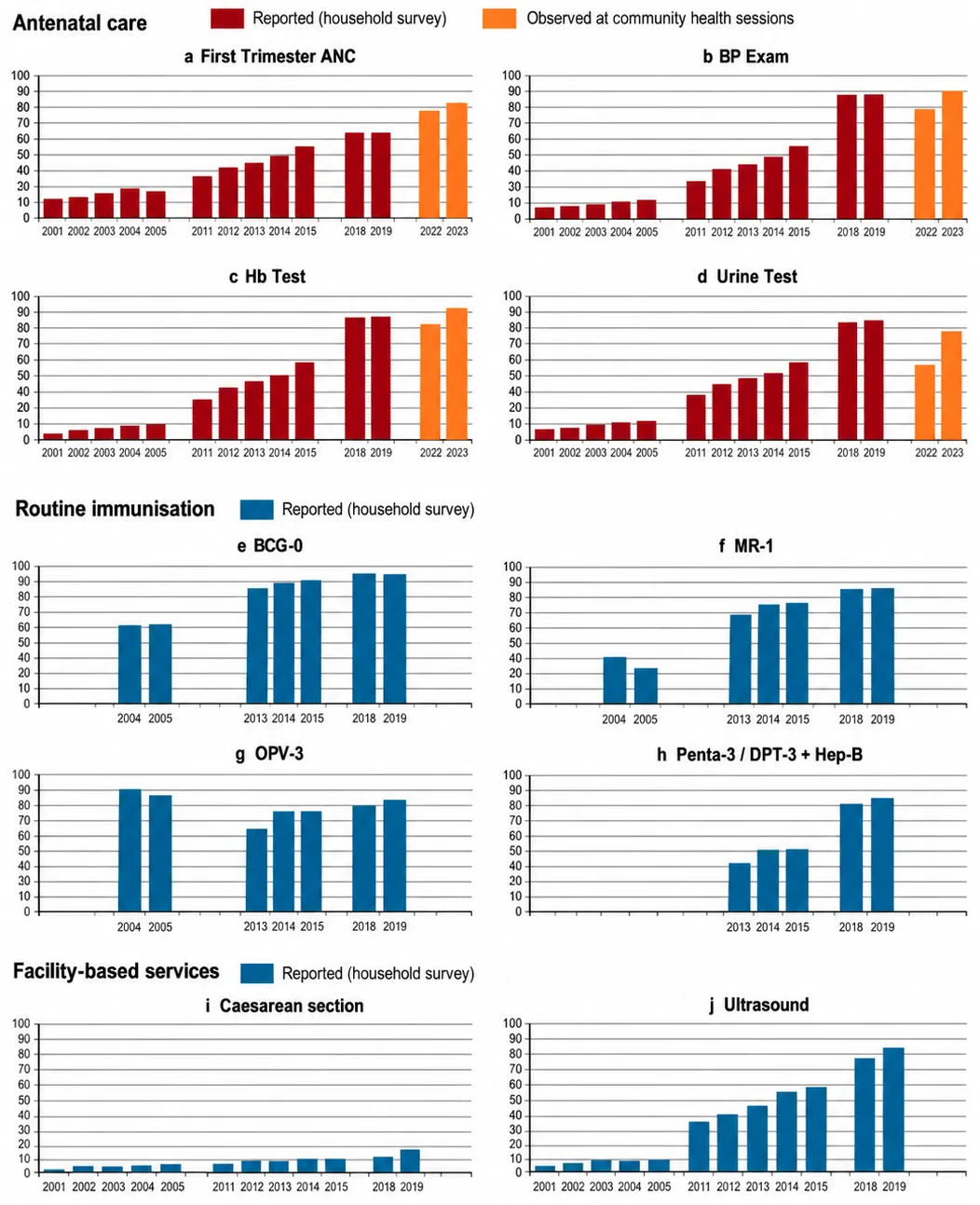
Antenatal care (ANC) content, routine immunisation completeness and facility-dependent maternity services in rural Uttar Pradesh, India, 2001–2023. Antenatal care: (a) first-trimester ANC; (b) BP examination; (c) Hb test; (d) urine test. Routine immunisation: (e) BCG-0; (f) MR-1; (g) OPV-3; (h) Penta-3/DPT-3+HepB. Facility-based services: (i) caesarean section; (j) ultrasound. Reported estimates are from the National Family Health Survey rounds 3, 4 and 5; antenatal indicators are among women with a live birth (most recent live birth only); immunisation indicators are among children aged 12–23 months at interview, plotted by the child’s year of birth. Panels (b)–(d) also show observed estimates from direct observation of community health sessions (VHNDs), 2022–2023, calculated as the mean of session-level proportions. BP, blood pressure; DPT, diphtheria-tetanus-pertussis; Hb, haemoglobin; HepB, hepatitis B; MR, measles-rubella; OPV, oral polio vaccine; Penta, pentavalent vaccine; VHNDs, Village Health and Nutrition Days.

#### Availability and readiness of community health sessions

Across the 61 133 sessions observed, most had functional equipment available for BP measurement (84.6%) and Hb testing (89.4%), and readiness for urine testing was lower (67.2%) (figure 1). All three improved steadily over the period, with monthly readiness rising from 60.7% to 93.2% for BP measurement, from 67.8% to 96.0% for Hb testing, and from 35.0% to 84.4% for urine testing. Availability of a DPT-equivalent vaccine and of OPV was consistently high, each reaching at least 90% of sessions in 21 of the 22 complete months observed (93.0% and 98.2% overall). BCG availability averaged 94.2%. Availability of MR was consistently lower but increased over time, peaking at 71.1% in July 2023.

### Educational inequality in service utilisation

Figure 4 shows pronounced education-related disparities in rural ANC/RI utilisation in 2001–2006, with higher coverage among women with 10+ years of education, particularly for content-qualified ANC and individual ANC components. For example, 1.9% of women with no education met the ANCq ≥8 threshold, compared with 23.9% of women with 10+ years of education. Over time, disparities narrowed across most ANC indicators as coverage increased across all education groups. A clear education gradient in full RI was evident in the earliest RI cohort (2004–2005). By 2018–2020, full RI had risen among children of women with no education (13.9% to 67.6%) and among those with 10+years of education (47.1% to 75.2%). Coverage of the DPT-equivalent series (DPT3/Penta3) and the measles-containing vaccine (measles/MMR/MR) followed similar patterns. OPV coverage was higher in 2004–2005 than in 2018–2020, whereas BCG coverage rose over the same period. BCG showed a marked education gradient in the earliest cohort that declined over time, whereas OPV was more evenly distributed across education levels throughout. Ultrasound use and caesarean section births both remained more common among women with higher education, but their inequality trajectories diverged. Relative inequality fell steeply for ultrasound, from a CIX of 0.375 (95% CI 0.288 to 0.461) in 2001–2006 to 0.190 (0.182 to 0.198) in 2011–2016, and to 0.059 (0.053 to 0.064) in 2018–2020. For caesarean birth, it fell only from 0.394 (0.250 to 0.538) to 0.257 (0.221 to 0.293), a reduction of about one third, leaving caesarean birth more concentrated among more educated women than any other indicator examined. Absolute inequality moved in the opposite direction for both facility-dependent services. The education SII rose from 0.228 (0.007 to 0.449) to 0.310 (0.286 to 0.335) for ultrasound and from 0.096 (−0.064 to 0.256) to 0.202 (0.040 to 0.363) for caesarean birth, whereas it fell from 0.473 (0.073 to 0.873) to 0.111 (0.085 to 0.136) for full RI and from 0.490 (0.166 to 0.814) to 0.068 (0.019 to 0.116) for any ANC. Education-related trends for the individual ANC and RI components are shown separately (see online supplemental 4). Parallel analyses using household wealth quintiles showed the same pattern (see online supplemental 1) (see online supplemental 2) (see online supplemental 3).

**Figure 4:**
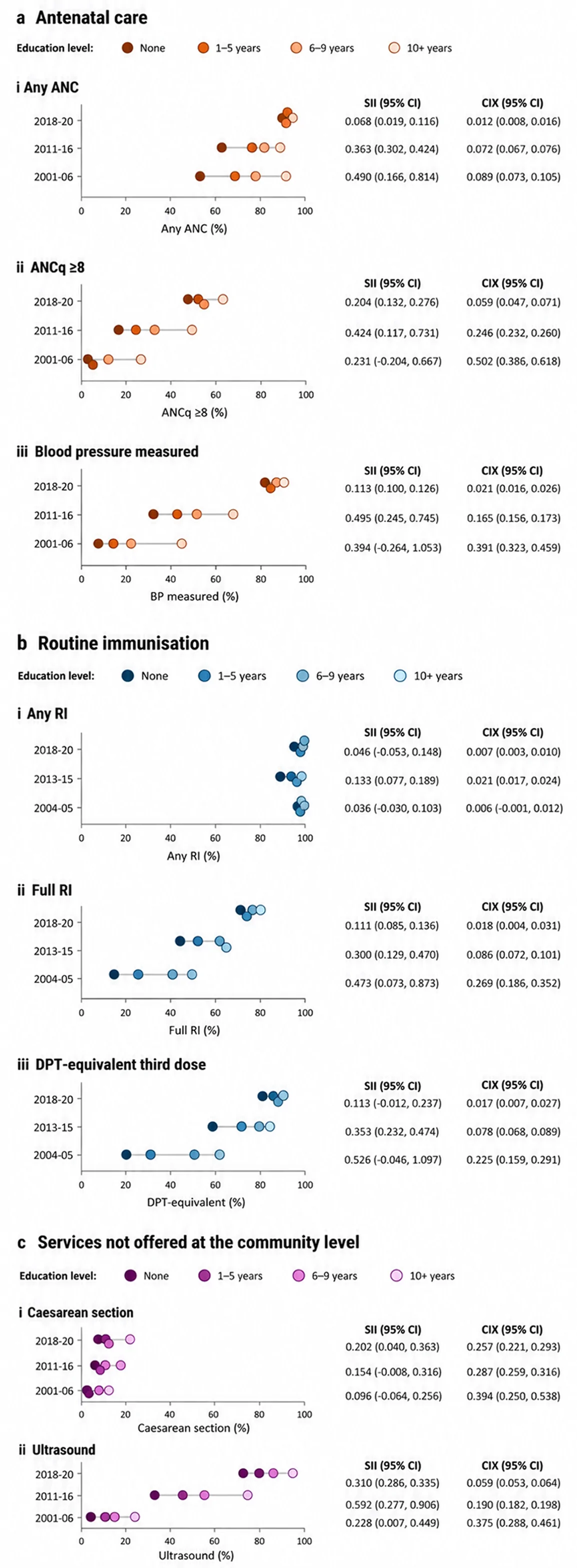
Education-related inequality in antenatal care (ANC), routine immunisation and facility-dependent maternity services in rural Uttar Pradesh, India. Coverage is shown by maternal education (none, 1–5, 6–9 and 10+ years of schooling) for each survey period. (a) Antenatal care: (i) any ANC; (ii) ANCq ≥8; (iii) BP measured. (b) Routine immunisation: (i) any RI; (ii) full RI; (iii) DPT-equivalent third dose. (c) Services not offered at the community level: (i) caesarean section; (ii) ultrasound. ANCq ≥8 denotes a content-qualified antenatal care score of at least 8. DPT-equivalent third dose is the third dose of pentavalent or DPT vaccine. SII, slope index of inequality (absolute); CIX, concentration index (relative). Positive values indicate higher coverage among more educated women (for immunisation, children of more educated mothers). ANCq, content-qualified antenatal care; BP, blood pressure; CIX, Concentration Index; DPT, diphtheria-tetanus-pertussis; RI, routine immunisation; SII, Slope Index of Inequality.

## Discussion

Rural communities frequently face barriers to timely primary care, including long travel distances, shortages of skilled providers and constraints in infrastructure and supplies,^24^ hindering universal health coverage. These barriers can contribute to both the non-use of services and to incomplete receipt of care when services are accessed.^25^ Community-based and outreach service delivery models that are reliably scheduled and adequately resourced are potential strategies to reduce geographic and opportunity-cost constraints in rural settings.^24,26^ In this repeated cross-sectional analysis of rural UP, we described long-term trends in the coverage, care content and education-related inequality of ANC and RI, services of a type typically delivered through monthly community health sessions. Any ANC became nearly universal, the proportion of women receiving content-qualified ANC (ANCq ≥8) increased more than 16-fold, and full RI coverage more than tripled. Improvements were largest among women with lower levels of education, coinciding with a narrowing of absolute and relative disparities, which must be interpreted in light of the changing educational composition of the population. These gains were strongest for content-qualified coverage measures, notably content-qualified ANC and completion of the RI schedule, rather than for ‘any-contact’ indicators that were already high early in the study period, which reinforces the importance of measuring care content and completeness rather than contact alone.^7^ At the same time, absolute education-related inequality persisted or widened for facility-dependent ultrasound and caesarean use, even as relative inequality narrowed for both. These changes occurred during a period when predictable community health sessions were scaled up alongside many other health-system changes.

Interpretation of these trends should be situated within the broader evolution of India’s health system between 2001 and 2020. This period coincided with major reforms under the National Rural Health Mission and the National Health Mission, including the expansion of the ASHA workforce, conditional cash transfers for facility births through Janani Suraksha Yojana and intensified immunisation campaigns such as Mission Indradhanush and Intensified Mission Indradhanush.^27,28^ These initiatives have been associated with increased maternal service utilisation and improved immunisation coverage in India.^27,28^ At the same time, fertility decline and rising female education may have increased households’ capacity to invest in maternal and child health and to act on health information.^29,30^ Because these shifts occurred concurrently with the expansion of the monthly community health sessions, the observed trends cannot be attributed to any single programme component, nor to the community health platform specifically. They are population-level changes that unfolded within a concurrently reforming health system.

Within this broader reform context, education-related inequality narrowed furthest for content-qualified antenatal and immunisation coverage, whereas absolute inequality persisted or widened for facility-dependent ultrasound and caesarean use. This descriptive differentiated pattern is consistent with the possibility that the type of service and where it can be delivered shape how improvements are distributed. Mechanisms consistent with this interpretation are well described in the literature. Geographic proximity and predictable service location and timing can reduce opportunity costs of care, including travel distance, transport expenses, lost wages and childcare constraints, which disproportionately affect households with fewer resources.^24,26^ Mobilisation and follow-up by community health workers can reduce informational and social barriers, facilitate early identification of pregnancies and support navigation of services.^31,32^ Integration of services within a single outreach session may increase the likelihood that women and children receive a minimum bundle of essential preventive interventions when they attend, linking immunisation contacts with key ANC components.^33,34^ Because the direct observations (2022–2023) postdate the survey trends, they are supportive and plausibility-enhancing rather than confirmatory. They show that community sessions in the later period were generally ready to deliver key ANC and RI inputs, but they cannot validate the mechanisms underlying the historical trends.

In contrast, widening absolute education gradients in ultrasound use and caesarean births underscore structural differences in the use of higher-level services. Because more-educated rural women may also live closer to towns, private providers, ultrasound services and surgical capacity, these gradients likely reflect geographic and facility availability within rural UP as much as maternal education itself; restricting the sample to rural areas does not remove this within-rural variation. These patterns were consistent across both education-based and wealth-based stratifications and are aligned with well-described ‘inverse equity’ dynamics, in which technology-intensive or facility-dependent services initially diffuse among socioeconomically advantaged groups unless access barriers are explicitly addressed.^15^ Ultrasound and caesarean delivery depend on facility infrastructure, surgical teams, anaesthesia, referral and transport systems and depending on the context, financing arrangements and the availability of services in the private sector.^35–37^ In interpreting these gradients, it is important to distinguish absolute and relative inequality. As ultrasound use becomes common across all education groups, relative inequality can decline while sizeable absolute gaps persist; for caesarean births, rising overall rates can coincide with increasing absolute differences even if relative inequality changes less. Because the data do not capture clinical need or indication, education-related differences in ultrasound and caesarean use are best described as utilisation gradients rather than inequities in appropriate care, and higher use cannot be equated with better care. Lower use among less-educated women may reflect underuse of medically indicated caesareans, while higher use among more advantaged women may reflect appropriate use, overuse or greater private-sector availability influenced by provider incentives, convenience, perceived safety and limited regulatory oversight in parts of the private sector.^35,38^ These data cannot distinguish among these explanations. Caesarean birth should therefore not be interpreted in the same way as full immunisation, for which higher coverage has a clearer positive meaning. Consequently, persistent or widening education-related inequality in facility-dependent ultrasound and caesarean use can coexist with narrowing inequality in content-qualified antenatal and immunisation coverage, reflecting the distinct resource requirements and governance structures of different components of primary care.

Three distinct changes should be interpreted separately. First, average coverage rose for most indicators. Second, coverage rose within each education stratum, including among women with no formal education. Third, the educational composition of the population changed markedly, and the proportion of women with no education fell from about 70% to under 30% across rounds. Because the education strata do not represent the same populations over time, a narrowing gradient cannot be read as the same disadvantaged group ‘catching up’. The women with no education in the later rounds are a smaller and potentially more selected group, and part of the narrowing may reflect this changing composition. For near-universal contact indicators (any ANC, any RI), ceiling effects among more-educated women also compress the gradient, which does not apply to content-qualified ANC or full RI, where more-educated women’s baseline coverage was well below any ceiling. Education-related trends for the individual ANC and RI components are shown separately (see online supplemental 4). Parallel analyses using household wealth quintiles showed the same pattern (see online supplemental 1) (see online supplemental 2) (see online supplemental 3), indicating that this reduction in inequality is not solely a compositional artefact of the shrinking no-education group, though education and wealth are correlated and this cannot fully exclude it.

This study has several strengths, including the use of large, state-representative survey data spanning two decades to assess trends in primary care contact, content-qualified care (ANCq) and education-related inequalities. The direct-observation component provides a large-scale, current-state picture of whether community sessions are equipped to deliver key ANC and immunisation inputs reliably; rather than validating historical survey trends, it establishes a contemporary readiness baseline that motivates continued monitoring of whether recent gains are sustained. Several limitations should be considered. Repeated cross-sectional analyses describe population-level changes over time but do not establish causal effects of community health sessions or any single programme component. We could not measure the intensity, functioning, fidelity or a counterfactual for community health sessions, and did not adjust for potential confounders. Over the study period, there were substantial concurrent changes in maternal education, fertility, socioeconomic conditions, health infrastructure and broader health-system reforms and we cannot isolate the independent contribution of community health platforms to the observed trends. As noted, changing educational composition and, for near-universal indicators, ceiling effects may partly explain the narrowing gradients. Survey-based measures are subject to recall and social desirability bias. Recall may also differ by education level, which could contribute to the observed gradients and their narrowing. Although ANCq was developed for use in household survey data, misclassification of specific components remains possible. Each survey applies a fixed lookback window to a moving interview date, and only each woman’s most recent live birth is analysed; thus, the number of births contributing to each year varies substantially, with the lowest counts at the start and end of each survey round. Annual estimates should therefore be read as describing the shape of change over time rather than as precise year-specific population estimates. Ultrasound and caesarean indicators capture utilisation without clinical indication and cannot be interpreted as appropriate or high-quality care. The surveys do not record where the components of content-qualified ANC or the vaccinations counted in full RI were received; these are population coverage indicators spanning all providers, and only the separately reported community-level ANC and community-level RI indicators identify care received at community health sessions. Our contrast between community-deliverable and facility-dependent services therefore reflects the type of service and its delivery requirements, not a measured delivery channel. Session-level readiness indicates whether an observed session was prepared to deliver services; it does not measure whether eligible women and children could reach that session or obtain timely, continuous care and should not be read as a measure of access. Observation-based readiness and service-delivery measures reflect a later-period snapshot and are supportive rather than confirmatory of readiness across the survey period. We intentionally segmented the survey analyses to reduce COVID-19-related disruptions, but residual effects cannot be fully ruled out.

In conclusion, between 2001 and 2020 in rural UP, coverage of antenatal and child health services increased substantially, with the largest gains observed for content-qualified ANC (ANCq ≥8) and for completion of the full RI schedule. These improvements were greatest among women with lower education, alongside narrower education-related inequalities for content-qualified ANC and full immunisation. In contrast, persistent or widening gradients were observed for ultrasound use and caesarean births, services that depend on higher-level facility capacity, referral and transport and often on private-sector availability and financing. Interpreted alongside large-scale observations of community-session readiness, these findings document substantial gains in content-qualified first-contact primary care and a narrowing of education-related inequality over a period of concurrent health-system change. Continued monitoring of care content alongside contact, and inequality alongside average coverage, is essential to ensure that progress towards universal health coverage reaches the households furthest from care.

## Supplementary material

10.1136/bmjph-2026-005227 online supplemental file 1

10.1136/bmjph-2026-005227 online supplemental file 2

10.1136/bmjph-2026-005227 online supplemental file 3

10.1136/bmjph-2026-005227 online supplemental file 4

## Data Availability

Data are available in a public, open access repository. Data are available on reasonable request.
