# Supplementary material for: Contact coverage, content of care and inequality in antenatal care and routine immunisation in rural Uttar Pradesh, India: a repeated cross-sectional analysis of household surveys and direct observations of community health sessions
Source: BMJ Public Health. 2026 Nov 2;4(4):e005227. doi: 10.1136/bmjph-2026-005227 (PMC13735361; doi:10.1136/bmjph-2026-005227)
Supplement: Supplementary file 1 [file bmjph-4-4-s001.pdf]

Supplementary 1. Antenatal care coverage and content-qualified care indicators (any ANC, ANCq ≥8, and blood pressure measurement) by maternal education and household wealth in rural Uttar Pradesh, India, by birth cohort.

	**Births in 2001-2006 (n=3045)**	**Births in 2011-2016 (n=22154)**	**Births in 2018-2020 (n=8669)**	**P value (across birth cohorts)**
**Any ANC**				
Women who received any ANC	62.8	74.6	91.1	<0.001
Maternal education	*P <0.001	*P <0.001	*P <0.001	
None	55.7	64.3	88.8	<0.001
1-5 years	69.9	76.2	90.6	<0.001
6-9 years	78.6	81.6	90.5	<0.001
10+ years	91.4	88.1	93.5	<0.001
Education slope index of inequality for ANC	P = 0.023<br>0.490 (0.166, 0.814)	P = 0.002<br>0.363 (0.302, 0.424)	P = 0.027<br>0.068 (0.019, 0.116)	
Education concentration index for ANC	0.089 (0.073, 0.105)	0.072 (0.067, 0.076)	0.012 (0.008, 0.016)	
Wealth quintiles	*P <0.001	*P <0.001	*P <0.001	
Q1 (poorest)	51.5	61.5	88.1	<0.001
Q2	61.6	76.4	91.8	<0.001
Q3	67.5	83.9	92.8	<0.001
Q4	82.9	88.5	92.6	<0.001
Q5 (wealthiest)	90.3	93.8	94.3	0.272
Wealth slope index of inequality for ANC	P = 0.011<br>0.385 (0.169, 0.602)	P <0.001<br>0.401 (0.340, 0.463)	P = 0.019<br>0.072 (0.023, 0.120)	
Wealth concentration index for ANC	0.094 (0.078, 0.109)	0.083 (0.078, 0.087)	0.013 (0.009, 0.017)	
**ANCq ≥8**				
Women who received an ANCq ≥8	5.0	25.4	50.6	<0.001
Maternal education	*P <0.001	*P <0.001	*P <0.001	
None	1.9	14.8	43.7	<0.001
1-5 years	4.1	21.8	47.8	<0.001
6-9 years	10.8	29.5	50.1	<0.001
10+ years	23.9	44.9	57.6	<0.001
Education slope index of inequality for ANCq	P = 0.150<br>0.231 (-0.204, 0.667)	P = 0.027<br>0.424 (0.117, 0.731)	P = 0.007<br>0.204 (0.132, 0.276)	
Education concentration index for ANCq	0.502 (0.386, 0.618)	0.246 (0.232, 0.260)	0.059 (0.047, 0.071)	
Wealth quintiles	*P <0.001	*P <0.001	*P <0.001	
Q1 (poorest)	1.4	13.7	40.7	<0.001
Q2	1.9	21.5	50.2	<0.001
Q3	4.4	31.7	55.5	<0.001
Q4	14.6	43.3	59.5	<0.001
Q5 (wealthiest)	37.2	63.3	67.2	<0.001
Wealth slope index of inequality for ANCq	P = 0.210<br>0.184 (-0.184, 0.551)	P = 0.027<br>0.455 (0.099, 0.810)	P = 0.001<br>0.284 (0.206, 0.362)	
Wealth concentration index for ANCq	0.555 (0.434, 0.676)	0.275 (0.261, 0.289)	0.087 (0.075, 0.099)	
**Blood pressure measurement**				

Women who received a blood pressure measurement	11.1	44.0	85.2	<0.001
Maternal education	*P <0.001	*P <0.001	*P <0.001	
None	5.7	31.2	81.3	<0.001
1-5 years	12.3	41.3	83.4	<0.001
6-9 years	19.9	50.0	85.6	<0.001
10+ years	42.4	65.7	88.9	<0.001
Education slope index of inequality for blood pressure measurement	P = 0.124<br>0.394 (-0.264, 1.053)	P = 0.014<br>0.495 (0.245, 0.745)	P <0.001<br>0.113 (0.100, 0.126)	
Education concentration index for blood pressure measurement	0.391 (0.323, 0.459)	0.165 (0.156, 0.173)	0.021 (0.016, 0.026)	
Wealth quintiles	*P <0.001	*P <0.001	*P <0.001	
Q1 (poorest)	4.1	29.0	80.4	<0.001
Q2	6.7	41.7	86.1	<0.001
Q3	13.2	52.7	86.6	<0.001
Q4	26.7	65.9	89.2	<0.001
Q5 (wealthiest)	53.1	80.6	91.9	<0.001
Wealth slope index of inequality for blood pressure measurement	P = 0.107<br>0.312 (-0.124, 0.747)	P = 0.007<br>0.535 (0.276, 0.795)	P = 0.010<br>0.124 (0.057, 0.192)	
Wealth concentration index for blood pressure measurement	0.427 (0.357, 0.497)	0.187 (0.178, 0.195)	0.024 (0.019, 0.029)	

Notes. ANC indicators are measured among women reporting their most recent live birth within the five years preceding NFHS-3 and NFHS-4 and the two years preceding NFHS-5; birth cohorts are defined by the year of that most recent live birth.

ANCq score ranges from 0 to 10 and assigns points for contact with a skilled provider, first-trimester care initiation, number of ANC visits, and receipt of key service components, including blood pressure (BP) measurement, haemoglobin (Hb) testing, neonatal tetanus protection, and urine testing.

*P values compare proportions across categories of the characteristic within a birth cohort.

P values reported for slope index of inequality correspond to tests of deviation from zero within each birth cohort.

Slope indices represent absolute inequality.

Concentration indices represent relative inequality.
