# Supplementary material for: Contact coverage, content of care and inequality in antenatal care and routine immunisation in rural Uttar Pradesh, India: a repeated cross-sectional analysis of household surveys and direct observations of community health sessions
Source: BMJ Public Health. 2026 Nov 2;4(4):e005227. doi: 10.1136/bmjph-2026-005227 (PMC13735361; doi:10.1136/bmjph-2026-005227)
Supplement: Supplementary file 2 [file bmjph-4-4-s002.pdf]

Supplementary 2. Routine immunisation indicators (any RI, full RI, and Penta-3/DPT-3+Hep-B) by maternal education and household wealth among children aged 12-23 months in rural Uttar Pradesh, India, across NFHS survey rounds.

	**Children aged 12-23 months in 2004-2005 (n=839)**	**Children aged 12-23 months in 2013-2015 (n=5956)**	**Children aged 12-23 months in 2018-2020 (n=2794)**	**P value (across cohorts)**
**Any RI**				
Mothers of children who received any RI	97.5	93.2	97.4	<0.001
Maternal education	*P = 0.399	*P <0.001	*P <0.001	
None	97.0	89.1	94.5	<0.001
1-5 years	97.6	93.6	97.2	0.018
6-9 years	100.0	96.1	98.6	0.003
10+ years	98.6	97.7	98.3	0.534
Education slope index of inequality for any RI	P = 0.144<br>0.036 (-0.030, 0.103)	P = 0.009<br>0.133 (0.077, 0.189)	P = 0.179<br>0.047 (-0.053, 0.148)	
Education concentration index for any RI	0.006 (-0.001, 0.012)	0.021 (0.017, 0.024)	0.007 (0.003, 0.010)	
Wealth quintiles	*P = 0.419	*P <0.001	*P <0.001	
Q1 (poorest)	96.7	89.4	96.1	<0.001
Q2	97.3	93.4	96.5	0.001
Q3	97.6	96.7	98.3	0.139
Q4	100.0	96.7	99.3	0.009
Q5 (wealthiest)	100.0	97.5	99.2	0.233
Wealth slope index of inequality for any RI	P = 0.068<br>0.033 (-0.004, 0.070)	P = 0.002<br>0.112 (0.078, 0.146)	P = 0.012<br>0.045 (0.019, 0.071)	
Wealth concentration index for any RI	0.005 (-0.001, 0.011)	0.018 (0.014, 0.022)	0.007 (0.003, 0.010)	
**Full RI**				
Mothers of children who received full RI	20.4	51.7	72.2	<0.001
Maternal education	*P <0.001	*P <0.001	*P = 0.005	
None	13.9	42.8	67.6	<0.001
1-5 years	24.4	50.1	70.0	<0.001
6-9 years	38.9	59.5	72.1	<0.001
10+ years	47.1	61.8	75.2	<0.001
Education slope index of inequality for full RI	P = 0.037<br>0.473 (0.073, 0.873)	P = 0.017<br>0.300 (0.129, 0.470)	P = 0.003<br>0.111 (0.085, 0.136)	
Education concentration index for full RI	0.269 (0.186, 0.352)	0.086 (0.072, 0.101)	0.018 (0.004, 0.031)	
Wealth quintiles	*P <0.001	*P <0.001	*P <0.001	
Q1 (poorest)	13.4	42.5	65.8	<0.001
Q2	16.0	51.2	71.5	<0.001
Q3	28.1	58.4	75.0	<0.001
Q4	31.8	63.8	79.1	<0.001
Q5 (wealthiest)	51.6	65.9	77.4	<0.001
Wealth slope index of inequality for full RI	P = 0.052<br>0.291 (-0.004, 0.587)	P <0.001<br>0.295 (0.264, 0.325)	P = 0.003<br>0.168 (0.105, 0.231)	
Wealth concentration index for full RI	0.230 (0.148, 0.312)	0.088 (0.074, 0.102)	0.034 (0.021, 0.048)	
**Penta-3/DPT-3+Hep-B**				

Mothers of children who received Penta-3/DPT-3+Hep-B	27.5	66.8	81.4	<0.001
Maternal education	*P <0.001	*P <0.001	*P <0.001	
None	20.4	56.2	75.6	<0.001
1-5 years	29.3	66.9	80.4	<0.001
6-9 years	48.9	74.6	82.4	<0.001
10+ years	58.6	79.1	84.1	<0.001
Education slope index of inequality for Penta-3/DPT-3+Hep-B	P = 0.058<br>0.526 (-0.046, 1.097)	P = 0.006<br>0.353 (0.232, 0.474)	P = 0.060<br>0.113 (-0.012, 0.237)	
Education concentration index for Penta-3/DPT-3+Hep-B	0.225 (0.159, 0.291)	0.078 (0.068, 0.089)	0.017 (0.007, 0.027)	
Wealth quintiles	*P <0.001	*P <0.001	*P <0.001	
Q1 (poorest)	19.1	57.1	75.8	<0.001
Q2	24.9	67.5	79.8	<0.001
Q3	34.1	73.4	85.2	<0.001
Q4	42.4	77.9	86.2	<0.001
Q5 (wealthiest)	54.8	81.9	87.5	<0.001
Wealth slope index of inequality for Penta-3/DPT-3+Hep-B	P = 0.017<br>0.316 (0.106, 0.525)	P <0.001<br>0.301 (0.269, 0.332)	P = 0.002<br>0.156 (0.111, 0.201)	
Wealth concentration index for Penta-3/DPT-3+Hep-B	0.180 (0.115, 0.246)	0.069 (0.059, 0.079)	0.028 (0.018, 0.038)	

Notes: Routine immunisation indicators are measured among women whose most recent child was aged 12-23 months at the time of interview; time periods refer to the interview period in which the child was 12-23 months old.
Full RI includes BCG, OPV-3, a DPT-equivalent third dose, and MR. The DPT-equivalent is Penta-3 or DPT-3+Hep-B in NFHS-4 and NFHS-5; in NFHS-3 it is three doses of plain DPT, as neither pentavalent nor DPT-hepatitis B vaccine was in use in India at that time.
Any RI indicates receipt of at least one routine childhood vaccine.
* P values compare proportions across categories of the characteristic within each cohort.
P values reported for slope index of inequality correspond to tests of deviation from zero within each cohort.
Slope indices represent absolute inequality.
Concentration indices represent relative inequality.
