# Supplementary material for: Contact coverage, content of care and inequality in antenatal care and routine immunisation in rural Uttar Pradesh, India: a repeated cross-sectional analysis of household surveys and direct observations of community health sessions
Source: BMJ Public Health. 2026 Nov 2;4(4):e005227. doi: 10.1136/bmjph-2026-005227 (PMC13735361; doi:10.1136/bmjph-2026-005227)
Supplement: Supplementary file 3 [file bmjph-4-4-s003.pdf]

Supplementary 3. Facility-dependent maternity services (caesarean section and antenatal ultrasound) by maternal education and household wealth in rural Uttar Pradesh, India, by birth cohort.

**Characteristic**	**Births in 2001-2006 (n=3045)**	**Births in 2011-2016 (n=22 154)**	**Births in 2018-2020 (n=8669)**	**P value (across birth cohorts)**
**Caesarean section**				
Women who gave birth by caesarean section	2.9	7.9	11.9	<0.001
Maternal education	*P <0.001	*P <0.001	*P <0.001	
None	1.6	4.2	5.7	<0.001
1-5 years	2.2	6.3	8.8	<0.001
6-9 years	6.3	8.6	9.9	0.058
10+ years	9.8	15.5	19.3	<0.001
Education slope index of inequality for caesarean section	P = 0.122<br>0.096 (-0.064, 0.256)	P = 0.055<br>0.154 (-0.008, 0.316)	P = 0.033<br>0.202 (0.040, 0.363)	
Education concentration index for caesarean section	0.394 (0.250, 0.538)	0.287 (0.259, 0.316)	0.257 (0.221, 0.293)	
Wealth quintiles	*P <0.001	*P <0.001	*P <0.001	
Q1 (poorest)	1.0	3.3	5.5	<0.001
Q2	1.2	6.0	10.0	<0.001
Q3	3.0	10.5	13.0	<0.001
Q4	8.4	14.6	19.5	<0.001
Q5 (wealthiest)	15.0	24.8	32.0	<0.001
Wealth slope index of inequality for caesarean section	P = 0.143<br>0.090 (-0.055, 0.236)	P = 0.045<br>0.184 (0.012, 0.357)	P = 0.040<br>0.225 (0.019, 0.431)	
Wealth concentration index for caesarean section	0.496 (0.340, 0.652)	0.358 (0.328, 0.388)	0.296 (0.259, 0.333)	
**Antenatal ultrasound**				
Women who received an antenatal ultrasound	7.2	45.9	82.6	<0.001
Maternal education	*P <0.001	*P <0.001	*P <0.001	
None	3.9	30.7	71.5	<0.001
1-5 years	9.8	42.8	78.4	<0.001
6-9 years	13.7	52.7	84.1	<0.001
10+ years	22.0	72.0	92.5	<0.001
Education slope index of inequality for ultrasound	P = 0.047<br>0.228 (0.007, 0.449)	P = 0.015<br>0.592 (0.277, 0.906)	P <0.001<br>0.310 (0.286, 0.335)	
Education concentration index for ultrasound	0.375 (0.288, 0.461)	0.190 (0.182, 0.198)	0.059 (0.053, 0.064)	
Wealth quintiles	*P <0.001	*P <0.001	*P <0.001	
Q1 (poorest)	2.5	24.5	67.4	<0.001
Q2	4.3	44.4	85.7	<0.001
Q3	9.2	61.2	90.3	<0.001
Q4	14.6	73.6	94.0	<0.001
Q5 (wealthiest)	38.9	88.2	96.3	<0.001
Wealth slope index of inequality for ultrasound	P = 0.140<br>0.201 (-0.120, 0.523)	P = 0.001<br>0.724 (0.544, 0.903)	P = 0.010<br>0.369 (0.167, 0.572)	

Wealth concentration index for ultrasound	0.434 (0.343, 0.524)	0.242 (0.234, 0.250)	0.073 (0.067, 0.078)

Notes. Facility-based service indicators are measured among women reporting their most recent live birth within the five years preceding NFHS-3 and NFHS-4 and the two years preceding NFHS-5; birth cohorts are defined by the year of that most recent live birth.
*P values compare proportions across categories of the characteristic within a birth cohort.
P values reported for slope index of inequality correspond to tests of deviation from zero within each birth cohort.
Slope indices represent absolute inequality.
Concentration indices represent relative inequality.
