# Supplementary material for: Contact coverage, content of care and inequality in antenatal care and routine immunisation in rural Uttar Pradesh, India: a repeated cross-sectional analysis of household surveys and direct observations of community health sessions
Source: BMJ Public Health. 2026 Nov 2;4(4):e005227. doi: 10.1136/bmjph-2026-005227 (PMC13735361; doi:10.1136/bmjph-2026-005227)
Supplement: Supplementary file 4 [file bmjph-4-4-s004.pdf]

Supplementary 4. Education-related inequality in selected antenatal care components and routine immunisation components in rural Uttar Pradesh, India, across NFHS rounds.

a. Antenatal care

Education level: None; 1–5 years; 6–9 years; 10+ years

i. First Trimester ANC

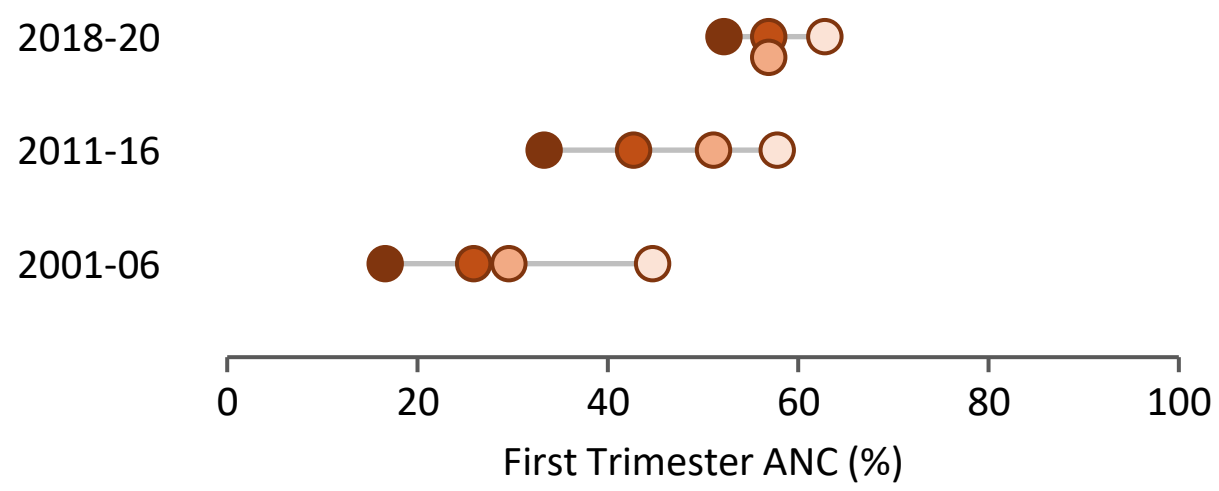

	SII (95% CI)	CIX (95% CI)
2018-20	0.153 (0.064, 0.241)	0.036 (0.025, 0.046)
2011-16	0.373 (0.299, 0.446)	0.125 (0.117, 0.134)
2001-06	0.339 (-0.040, 0.717)	0.164 (0.123, 0.204)

ii. Hb Test

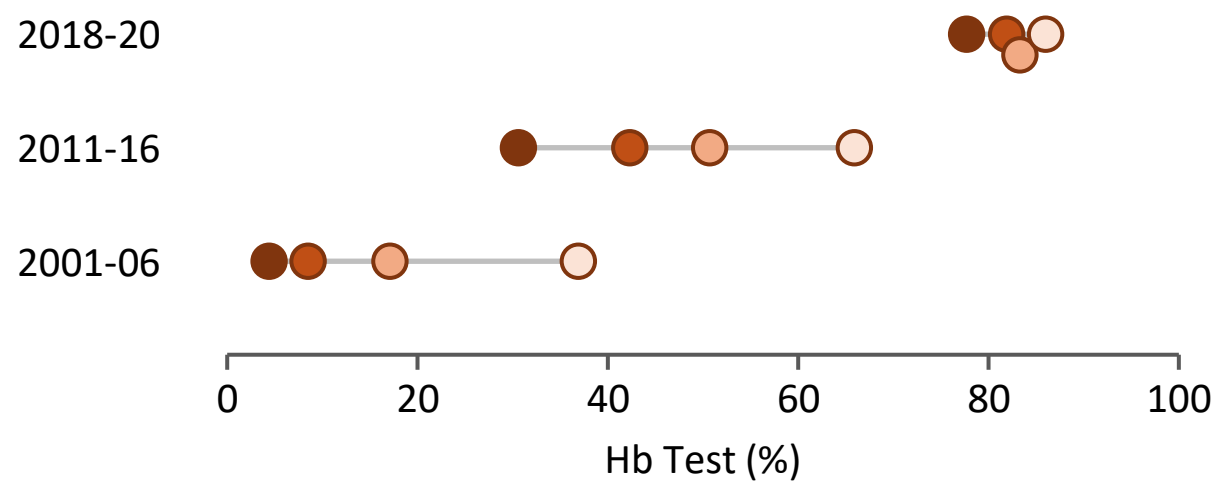

	SII (95% CI)	CIX (95% CI)
2018-20	0.119 (0.057, 0.182)	0.020 (0.014, 0.025)
2011-16	0.511 (0.307, 0.716)	0.173 (0.164, 0.181)
2001-06	0.342 (-0.282, 0.966)	0.414 (0.336, 0.492)

iii. Urine Test

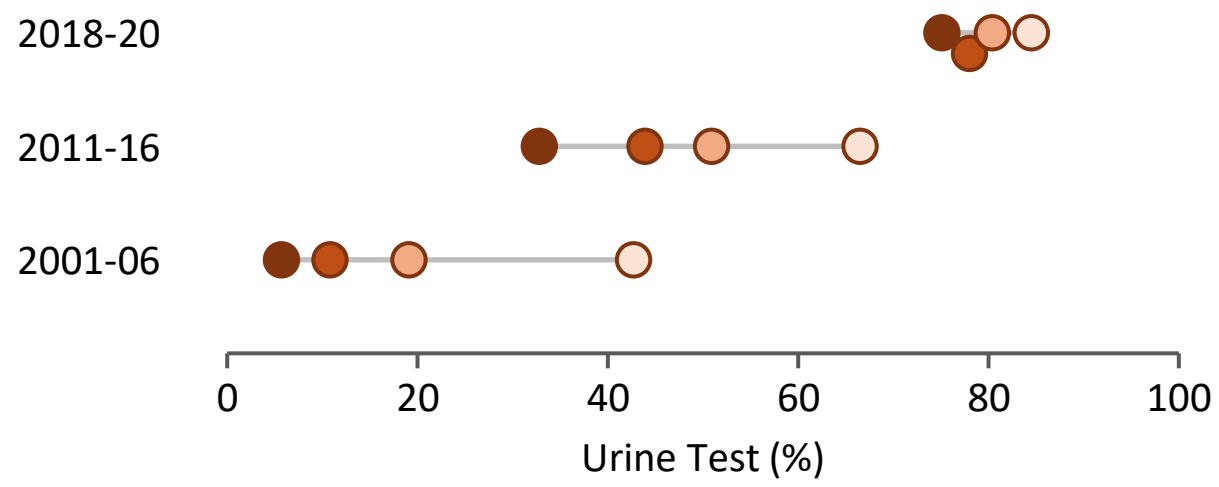

	SII (95% CI)	CIX (95% CI)
2018-20	0.138 (0.132, 0.145)	0.026 (0.020, 0.032)
2011-16	0.484 (0.257, 0.711)	0.159 (0.151, 0.167)
2001-06	0.384 (-0.335, 1.103)	0.401 (0.331, 0.470)

Notes. SII, slope index of inequality (absolute); CIX, concentration index (relative). BCG, dose of Bacille Calmette-Guérin vaccine, a single dose scheduled at birth; MR-1, first dose of measles-rubella vaccine; OPV-3, third dose of oral polio vaccine.

b. Routine immunisation

Education level: None · 1–5 years · 6–9 years · 10+ years

i. BCG-0

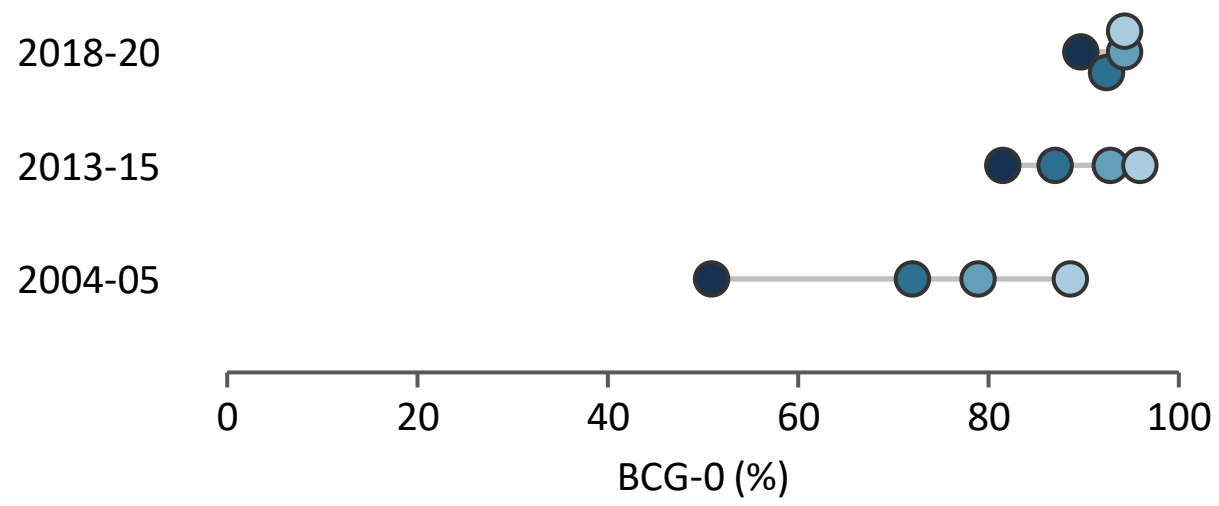

	SII (95% CI)	CIX (95% CI)
2018-20	0.060 (-0.045, 0.164)	0.009 (0.004, 0.015)
2013-15	0.220 (0.146, 0.294)	0.038 (0.032, 0.043)
2004-05	0.577 (0.422, 0.733)	0.111 (0.079, 0.144)

ii. MR-1

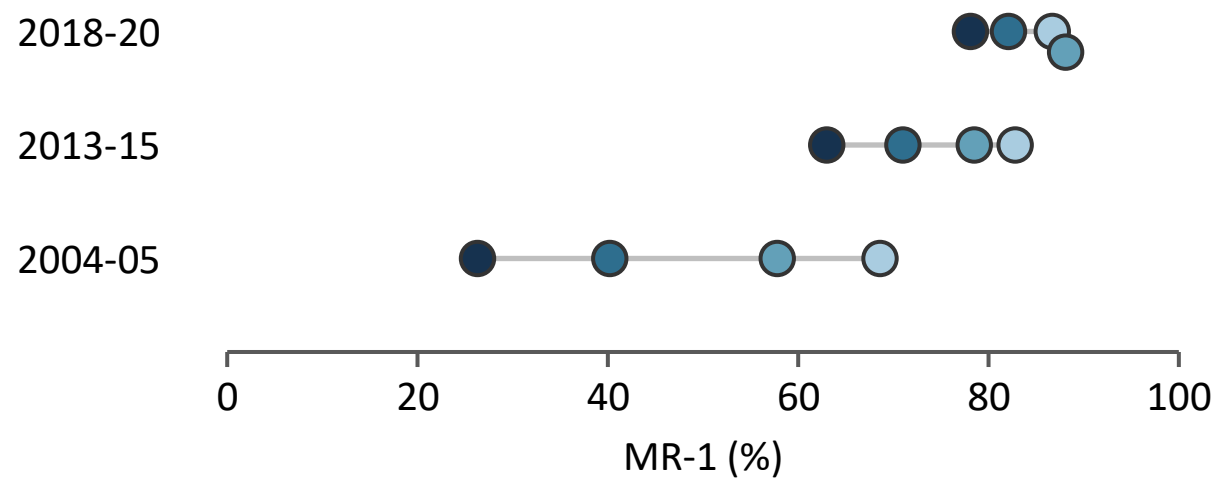

	SII (95% CI)	CIX (95% CI)
2018-20	0.115 (-0.135, 0.364)	0.018 (0.008, 0.027)
2013-15	0.302 (0.210, 0.395)	0.062 (0.053, 0.072)
2004-05	0.602 (0.116, 1.088)	0.198 (0.143, 0.253)

iii. OPV-3

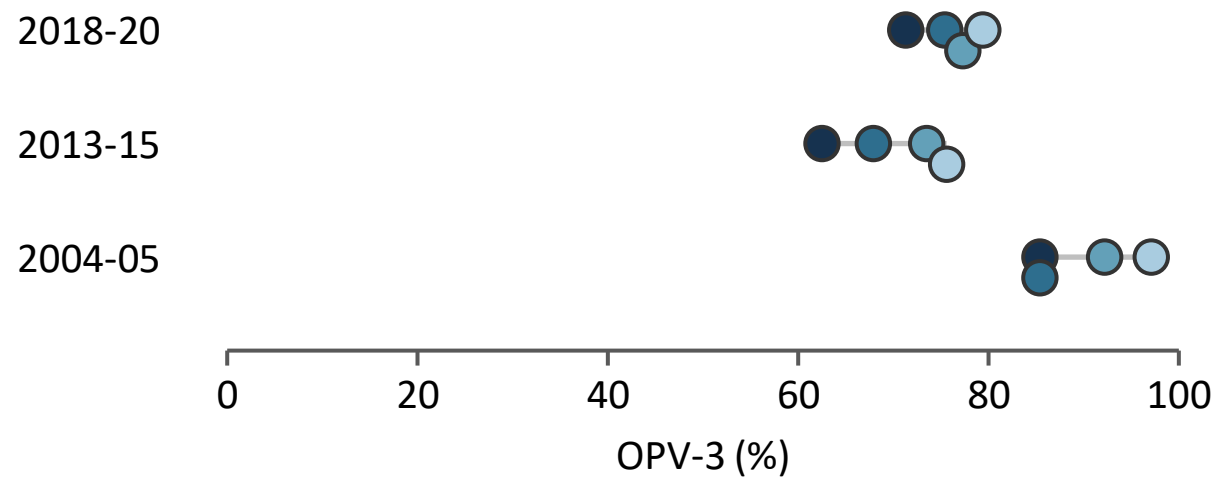

	SII (95% CI)	CIX (95% CI)
2018-20	0.111 (0.017, 0.205)	0.019 (0.007, 0.031)
2013-15	0.204 (0.113, 0.296)	0.044 (0.034, 0.054)
2004-05	0.134 (-0.130, 0.400)	0.013 (-0.002, 0.028)
